# Sir Henry Marsh

**Published:** 1861-03

**Authors:** 


SIR HENRY MARSH.—This distinguished physician died suddenly of apoplexy, at his residence in Dublin, on the 1st of December, 1860, in the seventieth year of his age. At the age of twenty-two, he commenced the study of surgery under the late Sir Philip Crampton, and pursued it with him for five years, when the loss of a portion of his right index finger, from a dissection wound, obliged him to turn his attention to medicine. In 1818 he received his medical degree in the University of Dublin, and two years later was appointed Assistant Physician to Steevens's Hospital. He lectured on the Practice of Medicine, in the Park Street Medical School, of which he was one of the founders, until 1827, when he was called to fill the same chair in the Royal College of Surgeons of Ireland. This position he held for five years, when he relinquished it on account of his increasing private practice. In 1837, Dr. Marsh was appointed Physician in Ordinary to the Queen for Ireland, and in 1839 he was created a baronet. One year later he was chosen President of the King's and Queen's College of Physicians, an office which he filled at various times. During the same year he became Senior Physician to Steevens's Hospital, a post which he retained up to within two or three years of his death.

For the last thirty years, Dr. Marsh occupied the highest position among the medical practitioners of Dublin. His contributions to medical science are to be found principally in the *Dublin Hospital Reports*, and in the *Dublin Journal of Medical Science.* At the time of his death he was engaged in publishing a work, entitled "Observations on Transmitted Affections of the Stomach, and on Disturbances of the Brain, which give rise to Somnambulism, Ecstacism, etc.," a part of which is already printed.

